# When “right” goes wrong: poor accuracy of calculated right adrenal vein aldosterone levels in subtyping primary aldosteronism

**DOI:** 10.1186/s42155-026-00769-6

**Published:** 2026-09-04

**Authors:** Saachi Datta, Robin Wang, Vivek Bhalla, Jehan Z. Bahrainwala, Susan Seav, Andrew C. Picel, David Wang, Matthew L. Hung

**Affiliations:** 1 Division of Interventional Radiology, Department of Radiology, Stanford University School of Medicine, 300 Pasteur Dr, Palo Alto, CA 94304 USA; 2 Division of Nephrology, Department of Medicine, Stanford University School of Medicine, 3180 Porter Drive, Palo Alto, CA 94304 USA; 3 Division of Endocrinology, Gerontology, and Metabolism, Department of Medicine, Stanford University School of Medicine, 300 Pasteur Dr, Palo Alto, CA 94304 USA; 4 Stanford Hypertension Center, Stanford University School of Medicine, 300 Pasteur Drive, Palo Alto, CA 94304 USA

**Keywords:** Primary aldosteronism, Adrenal vein sampling, Lateralization index

## Abstract

**Background:**

The purpose of this study is to externally validate a published ratio of left adrenal aldosterone to calculated-right adrenal aldosterone (Aldosterone_LAV_:cAldosterone_RAV_) proposed to determine disease subtype when right-sided adrenal vein sampling is unsuccessful.

**Materials and methods:**

This single-center retrospective study included 199 post-adrenocorticotropic hormone stimulation adrenal vein sampling cases (2016–2025). A selectivity index ≥ 5 was considered diagnostic and lateralization index ≥ 4 indicated unilateral disease. The cAldosterone_RAV_ was calculated as cAldosterone_RAV_ = [2 × Aldosterone_IVC_ × (Cortisol_LAV_/Cortisol_IVC_)]–Aldosterone_LAV_. The ratio of Aldosterone_LAV_:cAldosterone_RAV_ determined disease subtype; published thresholds were ≥ 3 for left-sided and ≤ 0.33 for right-sided disease. Intermediate values were deemed bilateral disease. Sensitivities and specificities of this metric were calculated using standard AVS interpretation as the gold standard reference. Receiver operating characteristic curve analysis performed on the Aldosterone_LAV_:cAldosterone_RAV_ ratio derived institution-optimized thresholds by maximizing the Youden’s *J *statistic.

**Results:**

Adrenal vein sampling was technically successful with determinate lateralization indices in 164 patients (41 left, 35 right, 88 bilateral). Using the published thresholds of 3 and 0.33, the Aldosterone_LAV_:cAldosterone_RAV_ ratio had an overall accuracy of 43.3% (95% CI 35.9–50.9%). Institution-optimized thresholds of ≥ 32.43 (left-sided) and ≤ 0.56 (right-sided) improved the accuracy to 65.9% (95% CI 58.3–72.7%). Using institution-optimized thresholds, the sensitivities/specificities for the calculated right-sided, left-sided, and bilateral disease were 88.6%/95.3%, 92.7%/63.4%, and 44.3%/93.4%, respectively.

**Conclusions:**

The Aldosterone_LAV_:cAldosterone_RAV_ ratio can help to rule in right-sided disease with high specificity, but otherwise has poor overall accuracy in disease subtyping, underscoring the importance of meticulous technique to achieve diagnostic samples during adrenal vein sampling.

**Supplementary Information:**

The online version contains supplementary material available at https://doi.org/10.1186/s42155-026-00769-6.

## Background

Primary aldosteronism is a common cause of secondary hypertension, occurring in up to 15% of hypertensive patients [1]. After primary aldosteronism is diagnosed, patients who desire surgery and are deemed candidates often will proceed to adrenal vein sampling (AVS) that determines whether aldosterone secretion is unilateral or bilateral. Unilateral disease leads to surgical intervention whereas bilateral disease requires lifelong medical management [1–4].

A common cause for technical failure during AVS is unsuccessful catheterization of the right adrenal vein, which can be challenging due to its small caliber and anatomical variation [3, 5–8]. Thus, AVS success rates are variable; the literature reports rates of successful bilateral cannulation ranging from 44 to 99% [3, 9, 10], and rates of successful right adrenal vein catheterization are lower compared to the left [11, 12].

When right-sided sampling fails, alternative ratios have been proposed to infer lateralization that utilize left-sided and inferior vena cava (IVC) cortisol and aldosterone values [13, 14]. Tuandam et al. recently proposed a novel method to determine unilateral disease with a published 92.6% sensitivity and 100% specificity [13]. Right-sided aldosterone concentration was calculated using only left adrenal vein and IVC measurements, assuming comparable cortisol production bilaterally. A ratio of left adrenal aldosterone: calculated-right adrenal aldosterone ratio (Aldosterone_LAV_:cAldosterone_RAV_) was used to determine disease subtype with cutoff values ≤ 0.33, ≥ 3, or intermediate values indicating right, left, and bilateral disease, respectively. Of the 108 patients with successful AVS in their study, 24 were excluded for imaging-discordant results between AVS and cross-sectional imaging.

Literature reports that 20–50% of patients with technically successful AVS procedures have discordant imaging [3, 5–8]. Further, it is known that AVS should be used as the gold standard because cross-sectional imaging assesses structure rather than function and there is a relatively high prevalence of non-functioning adrenal adenomas [7, 15–17]. By including only imaging-concordant cases (*n* = 68; nearly one-third of AVS cases in their cohort were excluded), the Tuandam et al. study results were from a homogenous subgroup which may lead to spectrum bias. The prospective utility of the calculated ratio has yet to be determined more broadly since it would not be known a priori whether AVS results will be concordant with cross-sectional imaging. As an initial retrospective, single-center study with a small cohort, the result may be prone to confounding and institution-specific effects. To our knowledge, the novel method proposed by Tuandam et al. has not been externally validated.

The present study aims to reproduce and evaluate the methods described on a larger cohort at a large tertiary center using technically successful AVS results as the reference standard independent of cross-sectional imaging findings.

## Materials and methods

**Fig. 1:**
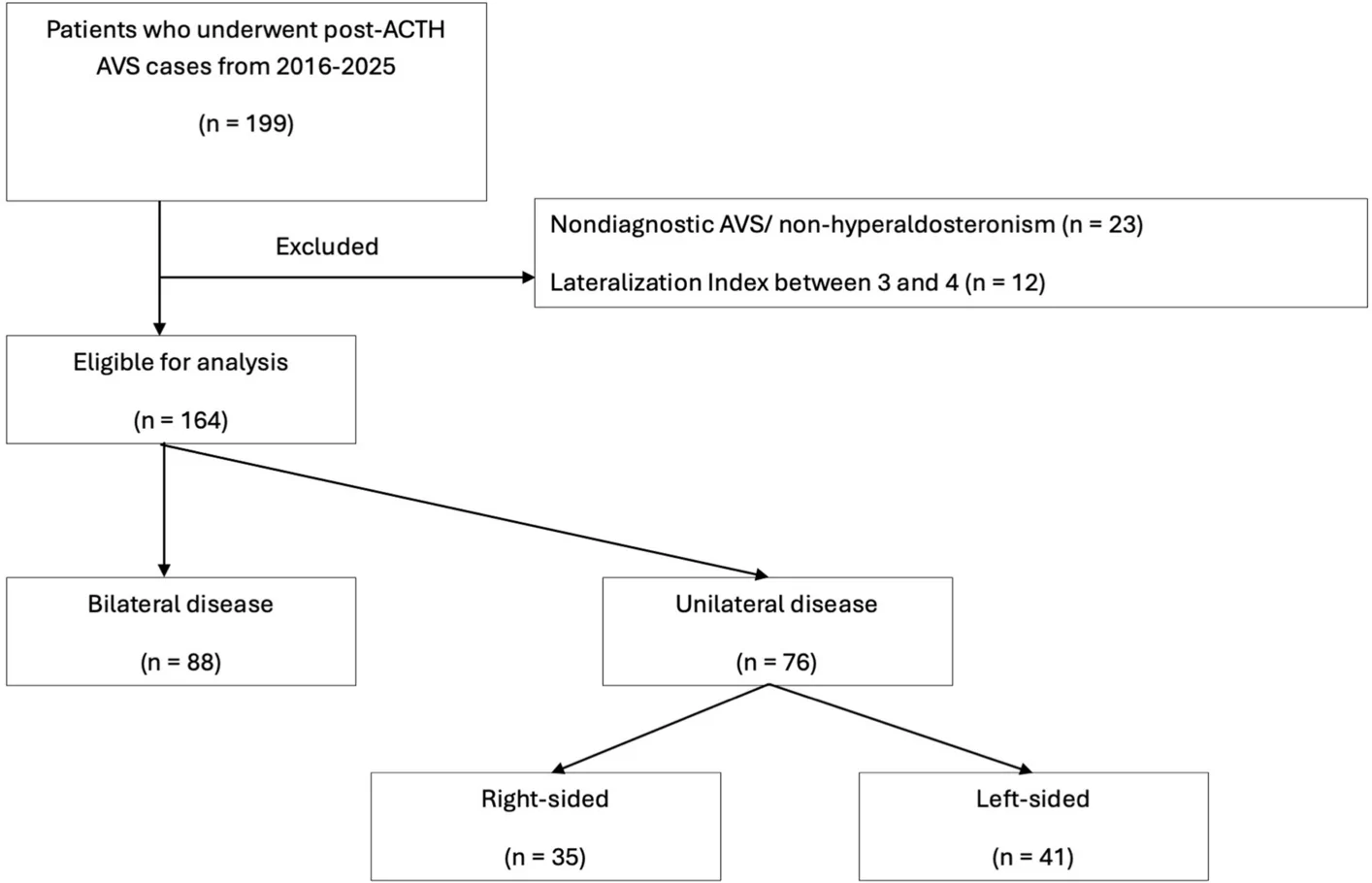
Flow diagram of eligible patients

A retrospective review of patients from a single-institution tertiary referral center was conducted. Institutional review board approval was obtained for this retrospective study which fully complied with the Health Insurance Portability and Accountability Act. An institutional tool for cohort discovery through the electronic medical records was used to identify 199 patients who underwent AVS between June 2016 and June 2025 and had selectivity index of ≥ 5, which was considered technically successful. Patients who underwent AVS for conditions involving hypersecretion of cortisol (i.e., Cushing syndrome, primary aldosteronism with mild autonomous cortisol secretion), had non-diagnostic AVS procedures, or technical failures were excluded. Patients with lateralization index values between 3 and 4 were considered indeterminate and also excluded. After applying exclusion criteria, the final cohort consisted of 164 patients who underwent successful AVS for primary aldosteronism (Fig. 1; mean age, 54.3 years; 66 females and 98 males). Patients were appropriately worked up for primary aldosteronism by the referring nephrologist or endocrinologist in accordance with societal guidelines [18, 19]. Data regarding baseline clinical characteristics, laboratory, imaging, and AVS results were obtained from the electronic medical record.

AVS was performed on an outpatient basis at a tertiary referral center by five board-certified interventional radiologists, with or without a trainee. Sampling was performed according to the standardized Mayo Clinic poststimulation sequential protocol [6]. All patients were infused with cosyntropin at 50 mcg/h starting at least 1 h before the procedure and continued throughout. Cortisol and aldosterone samples were obtained sequentially from the right adrenal vein, left adrenal vein, and inferior vena cava. Plasma aldosterone level and plasma renin activity were measured by liquid chromatography tandem mass spectrometry (Mayo Clinic Laboratories).

A selectivity index (Cortisol_adrenal_/Cortisol_IVC_) ≥ 5 was considered diagnostic. A lateralization index ([aldosterone-to-cortisol ratio]_higher adrenal_/[aldosterone-to-cortisol ratio]_lower adrenal_) ≥ 4 was indicative of unilateral disease, and ≤ 3 was considered bilateral.

The following equations from the methods proposed by Tuandam et al. were used under their assumption that cortisol production from the adrenal glands is equal bilaterally in order to calculate an aldosterone value from the right adrenal vein (cAldosterone_RAV_).

$$\mathrm{Dilution}\;\mathrm{Effect}={\mathrm{Cortisol}}_{\mathrm{LAV}}/{\mathrm{Cortisol}}_{\mathrm{IVC}}$$

$${\mathrm{Aldosterone}}_{\mathrm{IVC}}=0.5\ast({\mathrm{Aldosterone}}_{\mathrm{LAV}}+{\mathrm{Aldosterone}}_{\mathrm{RAV}})/\mathrm{Dilution}\;\mathrm{Effect}$$

$${\mathrm{c}\mathrm{A}\mathrm{l}\mathrm{d}\mathrm{o}\mathrm{s}\mathrm{t}\mathrm{e}\mathrm{r}\mathrm{o}\mathrm{n}\mathrm{e}}_{\mathrm{R}\mathrm{A}\mathrm{V}}=[2*\mathrm{A}\mathrm{l}\mathrm{d}\mathrm{o}\mathrm{s}\mathrm{t}\mathrm{e}\mathrm{r}\mathrm{o}\mathrm{n}\mathrm{e}*({\mathrm{C}\mathrm{o}\mathrm{r}\mathrm{t}\mathrm{i}\mathrm{s}\mathrm{o}\mathrm{l}}_{\mathrm{L}\mathrm{A}\mathrm{V}}/{\mathrm{C}\mathrm{o}\mathrm{r}\mathrm{t}\mathrm{i}\mathrm{s}\mathrm{o}\mathrm{l}}_{\mathrm{I}\mathrm{V}\mathrm{C}})]-{\mathrm{A}\mathrm{l}\mathrm{d}\mathrm{o}\mathrm{s}\mathrm{t}\mathrm{e}\mathrm{r}\mathrm{o}\mathrm{n}\mathrm{e}}_{\mathrm{L}\mathrm{A}\mathrm{V}}$$

$$\mathrm{Calculated}\;\mathrm{Lateralization}\;\mathrm{Index}={\mathrm{Aldosterone}}_{\mathrm{LAV}}/{\mathrm{cAldosterone}}_{\mathrm{RAV}}$$

A negative value cAldosterone_RAV_ was replaced by 37, the lowest aldosterone value in the nondominant side within this study’s data—replicating the approach proposed by Tuandam et al. to interpret negative cAldosterone_RAV_ values. The calculated disease subtype was determined with the index, Aldosterone_LAV_/cAldosterone_RAV_. The published cutoff values for this index of ≥ 3, ≤ 0.33, or intermediate indicating left-sided, right-sided, or bilateral disease, respectively, were used for the first set of analyses to determine specificity and sensitivity [13]. The reference standard for the subtype of disease was based on the routine interpretation of the AVS results. These criteria, that being a selectivity index ≥ 5 defining technical success, a lateralization index ≥ 4 defining unilateral disease, and ≤ 3 defining bilateral disease, were applied uniformly to all patients. Cross-sectional imaging was not incorporated into the reference standard, and imaging concordance was not an inclusion criterion. Receiver Operating Characteristic (ROC) curve and area under the curve (AUC) analyses were then performed to determine the center-specific optimal cutoff values. The institution-specific thresholds were derived using Youden’s* J* optimization in a one versus rest method, which identifies the ROC curve point with the best combined sensitivity and specificity. Overall accuracy was calculated as the percentage of cases in which the predicted classification exactly matched the reference classification. As a post hoc secondary analysis, accuracy was compared between imaging-concordant and imaging-discordant cases, with concordance defined as a dominant adrenal mass on the side of AVS lateralization for unilateral disease, and as either bilateral masses or no dominant mass for bilateral disease. All other combinations were classified as discordant. Because the Aldosterone_LAV_:cAldosterone_RAV_ ratio lacks a biologically established cutoff and disease subtypes were imbalanced in our cohort, Youden’s *J* provided an objective and prevalence-independent method for threshold selection. Scatterplots and ROC curves were generated using Matplotlib in Python 3.10 (Python Software Foundation) using NumPy, pandas, SciPy, scikit-learn, and statsmodels libraries.

## Results

We identified 199 patients who underwent AVS for primary aldosteronism; 82% were technically successful with a determinate lateralization index (i.e., ≥ 4 or ≤ 3), resulting in a cohort of 41 patients with left-unilateral, 35 with right-unilateral, and 88 with bilateral disease. Cross-sectional imaging was concordant with the AVS-determined subtype in 109 of 164 patients (66.5%). Baseline demographic and biochemical characteristics of these patients are in Table 1.

**Table 1:** Baseline characteristics of eligible patients

Characteristics	Left-sided (*n* = 41)	Right-sided (*n* = 35)	Bilateral (*n* = 88)
Age (years)	54.1 ± 11.7	52.9 ± 12.5	55.0 ± 12.0
Sex (female)	16 (39%)	6 (17%)	44 (50%)
Blood pressure medications (DDD)	4.76 ± 2.95	4.77 ± 2.62	4.10 ± 2.67
Hypertension severity: ^a^			
Normal	16 (39%)	14 (40%)	30 (34%)
Grade 1	14 (34%)	11 (31%)	28 (32%)
Grade 2	9 (22%)	8 (23%)	22 (25%)
Grade 3	2 (5%)	2 (6%)	8 (9%)
Spontaneous hypokalemia	38 (93%)	25 (71%)	43 (49%)
Plasma aldosterone concentration (ng/dL)	39.9 ± 37.3	28.8 ± 30.9	22.5 ± 19.9
Plasma renin activity < 0.6 ng/mL/hour	18 (44%)	13 (37%)	45 (51%)
Aldosterone: Renin Ratio	(n = 23)	(n = 22)	(n = 43)
20 ≤ ARR < 30	2	4	5
30 ≤ ARR < 40	1	2	5
ARR ≥ 40	17	15	20
Confirmatory testing for primary aldosteronism performed	24 (59%)	20 (57%)	59 (67%)
Adrenal mass on cross sectional imaging			
No imaging	0 (0%)	0 (0%)	0 (0%)
No mass	7 (17%)	6 (17%)	46 (52%);
Unilateral mass	33 (80%)	26 (74%)	35 (40%)
Bilateral mass	1 (2%)	3 (9%)	7 (8%)
Imaging concordance with AVS results	31 (76%)	25 (71%)	53 (60%)

Data are presented as mean ± SD or *n* (%) as appropriate

*DDD* defined daily dose [20]

^a^According to the 2018 European Society of Cardiology and European Society of Hypertension Guidelines [8]

**Fig. 2:**
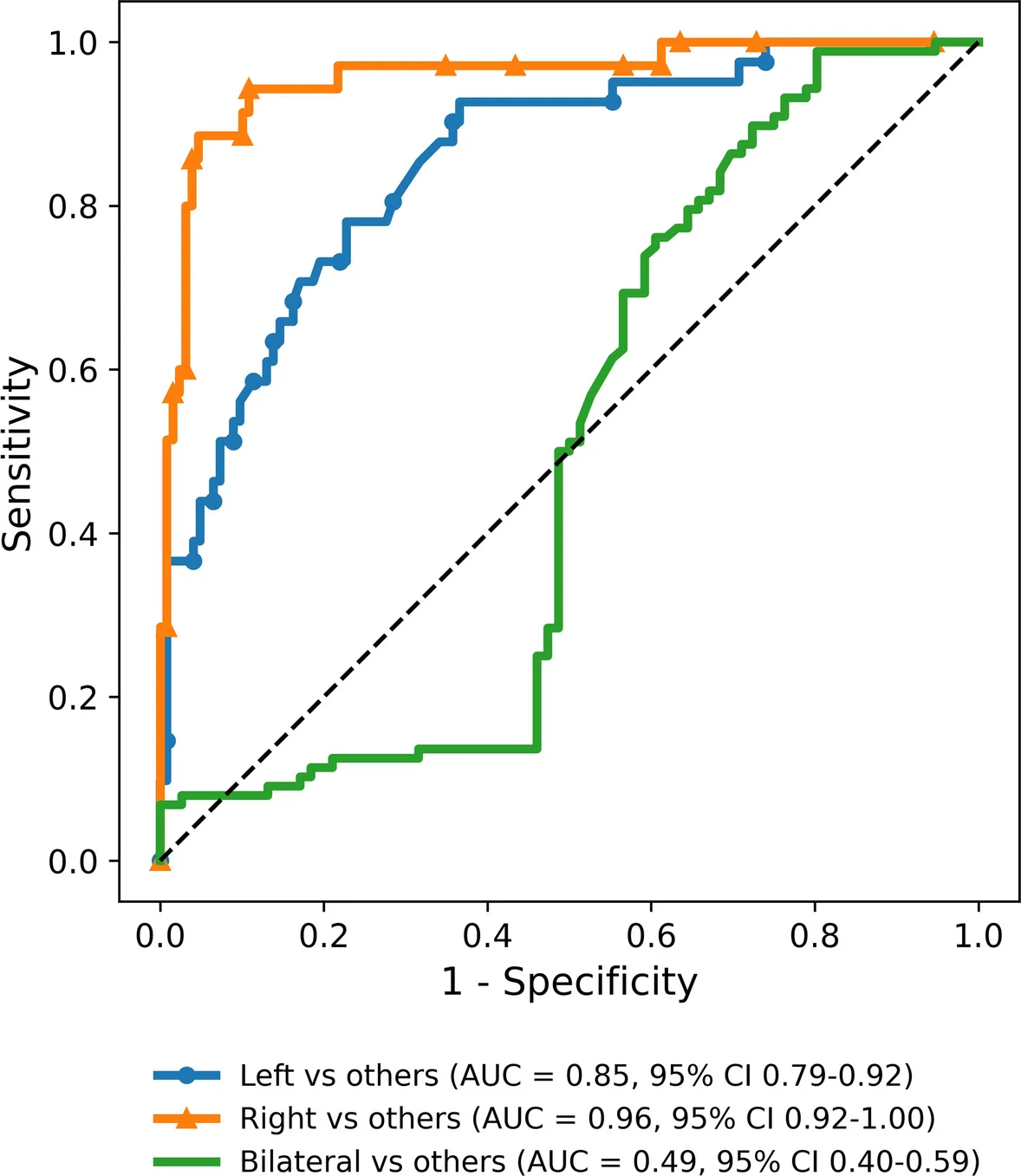
Receiver operating characteristic (ROC) Curves for lateralization threshold optimization. ROC curves showing performance of Aldosterone_LAV_:cAldosterone_RAV_ for classifying left, right, and bilateral disease on AVS. AUC values with 95% CIs are provided. Optimal institution-specific thresholds were derived using Youden’s *J* statistic

Using the originally published thresholds of ≥ 3 for left-sided disease and ≤ 0.33 for right-sided disease, 71 of 164 cases were correctly classified: 39 left-sided, 25 right-sided, and 7 bilateral*.* The Aldosterone_LAV_:cAldosterone_RAV_ ratio prediction had an overall accuracy of 43.3% (95% CI 35.9–50.9%) for determining disease subtype and could identify left-sided disease with 95.1% sensitivity and 35% specificity, right-sided disease with 71.4% sensitivity and 96.9% specificity, and bilateral disease with 8.0% sensitivity and 88.2% specificity. The AUC for left-sided disease was 0.85 (95% CI 0.79–0.92) compared to right-sided disease which was 0.96 (95% CI 0.92–1.00). The AUC for bilateral disease was 0.49 (95% CI 0.40–0.59). Exploratory optimal institution-specific thresholds were derived using ROC curve analysis and the Youden’s* J *statistic within this cohort. For left-sided disease, the threshold was ≥ 32.43, while for right-sided disease it was ≤ 0.56 (Fig. 2).

**Fig. 3:**
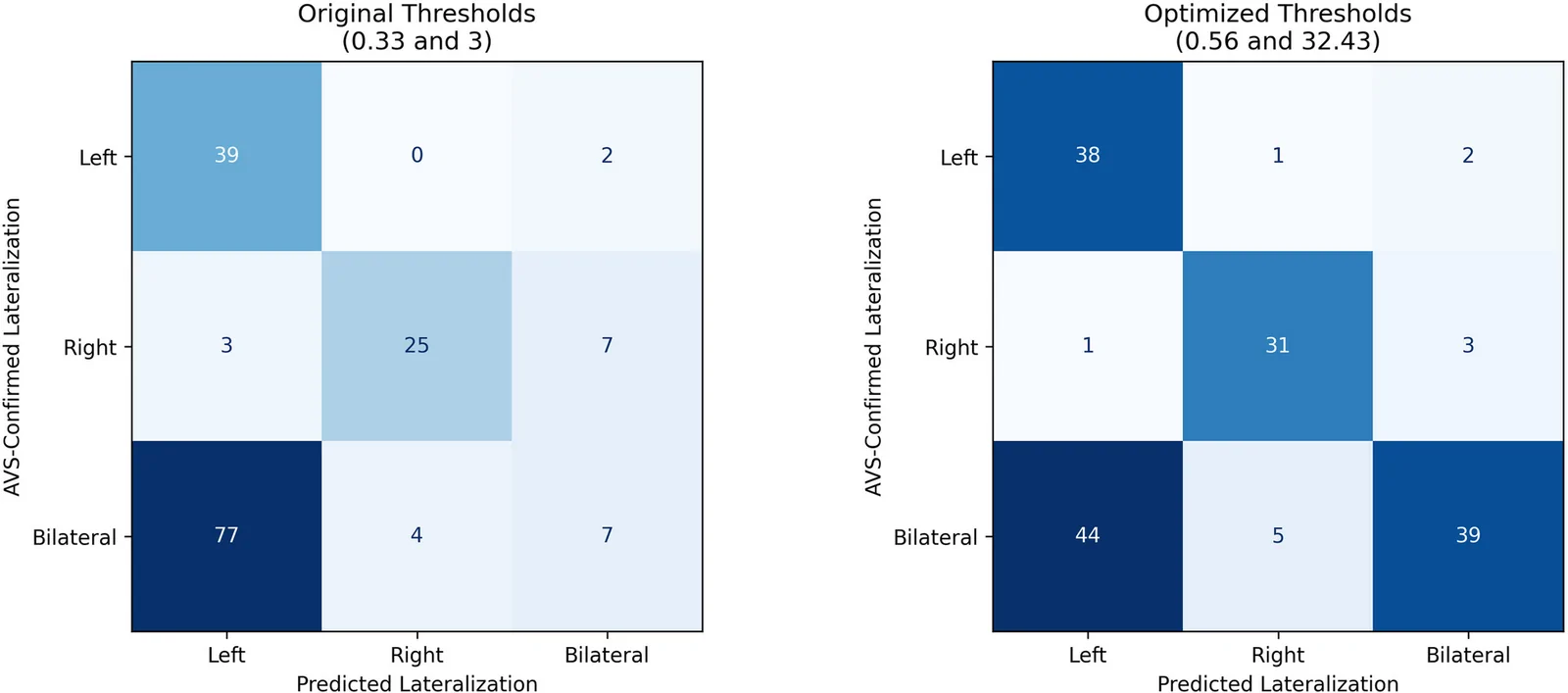
Confusion matrices for subtype classification using the original and optimized calculated-index thresholds. Rows show AVS-confirmed lateralization, columns show predicted lateralization, and darker cells indicate greater case counts

**Fig. 4:**
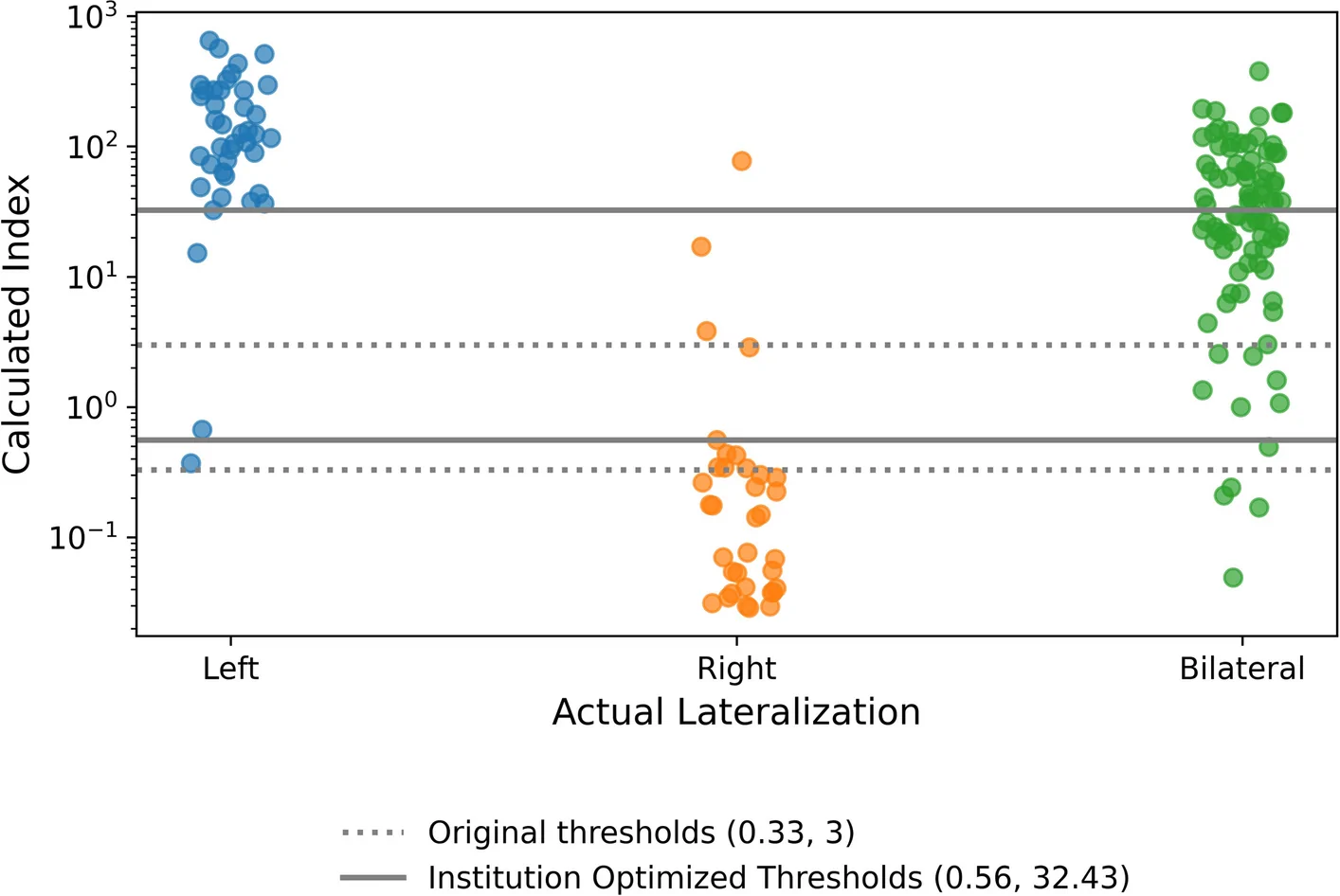
Scatterplot of Calculated Index Aldosterone_LAV_:cAldosterone_RAV_ by actual subtype (AVS results) Scatterplot of Aldosterone_LAV_:cAldosterone_RAV_ values for patients with AVS-confirmed left-sided, right-sided, or bilateral disease. Dotted lines mark published thresholds (0.33, 3.0); solid lines mark institution-optimized thresholds (0.56, 32.43). *Y*-axis shown on a log scale

Using the institution-optimized thresholds, 108 of 164 cases were correctly classified: 38 left-sided, 31 right-sided, and 39 bilateral (Fig. 3); the overall accuracy of the Aldosterone_LAV_:cAldosterone_RAV_ ratio for disease subtyping improved to 65.9% (95% CI 58.3–72.7%). With the optimized left-sided disease threshold of 32.43, sensitivity for left-sided disease was 92.7% and specificity was 63.4%. With the optimized right-sided threshold of 0.56, sensitivity for right-sided disease was 88.6% and specificity was 95.3%. For bilateral disease, sensitivity was 44.3% and specificity was 93.4%. The published and institution-optimized thresholds are visualized on the scatterplot of Aldosterone_LAV_:cAldosterone_RAV_ values plotted against the actual disease subtype according to AVS results (Fig. 4). The sensitivities and specificities for the Aldosterone_LAV_:cAldosterone_RAV_ ratio in determining disease subtype using published and institution-optimized thresholds are summarized in Table 2.

**Table 2:** Performance metrics of published and institution-optimized thresholds

	Published threshold sensitivity	Published threshold specificity	Institution-optimized threshold sensitivity	Institution-optimized threshold specificity
Left	0.951 (0.835–0.994)	0.350 (0.266–0.441)	0.927 (0.801–0.985)	0.634 (0.543- 0.719)
Right	0.714 (0.537–0.854)	0.969 (0.923- 0.991)	0.886 (0.733–0.968)	0.953 (0.902–0.983)
Bilateral	0.080 (0.033- 0.157)	0.882 (0.787–0.944)	0.443 (0.337- 0.553)	0.934 (0.853–0.978)

Data are presented as value (95% confidence interval). Published thresholds were ≤ 0.33 and ≥ 3. Institution-optimized thresholds were ≤ 0.56 and ≥ 32.43

Accuracy was mildly higher among imaging-concordant than imaging-discordant cases (47.7% vs 34.5% with the published thresholds), varying primarily with the proportion of bilateral disease, reaching 84.2% among unilateral cases alone and 8.0% among bilateral cases alone (Supplementary Table S1).

## Discussion

Treatment decisions for patients with primary aldosteronism often hinge on AVS results to determine whether surgical or medical management is appropriate [17]. Cannulation of the right adrenal vein remains technically challenging due to anatomical variation and size with higher failure rates compared to the left adrenal vein [2, 17, 21–23]. Tuandam et al. proposed a novel and creative method of calculating right aldosterone using a set of simple equations that were then compared with left-sided aldosterone levels to determine unilateral or bilateral disease based on optimized cutoff values. The published sensitivity and specificity for determining unilateral disease were 92.6% and 100% respectively, making it a potentially powerful tool in case of sampling failure of the right adrenal vein [13]. The current study sought to externally validate this approach and assess its reproducibility while broadening the inclusion criteria to better inform the prospective utility of this calculated metric.

Our study comprised a larger, more representative cohort (*n* = 164), over twice the size of the original dataset, and with more than half exhibiting bilateral disease (*n* = 88). Moreover, lateralized cases were balanced (left = 41 patients, right = 35 patients). This cohort also included both imaging-concordant and imaging-discordant patients, using standard AVS interpretation as the reference standard. The above inclusions and distributions were important for creating a balanced, unbiased cohort to test the calculated indices proposed by Tuandam et al. Despite these methodological strengths, both the original and institution-optimized thresholds yielded poor overall accuracy for disease subtyping. Using the original cutoff values proposed by Tuandam et al. (0.33, 3), the overall diagnostic accuracy in our cohort was only 43.3% (95% CI 35.9–50.9%). Sensitivity was high for left-sided disease, but was more limited for right-sided and bilateral disease (71.4% right, 95.1% left, 8.0% bilateral). Notably, performance for bilateral disease was particularly poor, with an AUC of 0.49, indicating essentially no discriminatory ability and limiting the utility of this calculated index for identifying patients who should be managed medically. Specificity was similarly limited (35.0% left, 88.2% bilateral). However, specificity for right-sided disease was relatively high (96.9%, 95% CI 92.3–98.8%), suggesting that a right-lateralized result may still have clinical utility. If right AVS fails and the proposed metric strongly suggests right dominance, clinicians may use this as supportive evidence for potential surgical candidacy.

Using our institution-optimized thresholds of 0.56 and 32.43, the overall accuracy improved to 65.9% (95% CI 58.3–72.7%) and bilateral disease sensitivity had a substantial increase from 8.0 to 44.3%, indicating that institution-specific recalibration can partially enhance model performance. Institution thresholds were derived and evaluated within the same cohort and should therefore be considered exploratory. The large shift in the optimized left-sided threshold was primarily driven by the handling of negative cAldosterone_RAV_ values which were replaced by an institution-derived constant. Because this substitution directly scales the calculated ratio, the resulting threshold is inherently institution- and assay-dependent; for example, changing the constant from 37 to 100 shifted the optimal cutoff from 32.43 to 12.00 without changing overall accuracy. This finding underscores that, if a calculation-based method were to be adopted, each institution should derive its own thresholds, which would require further validation in an independent cohort before clinical application, introducing additional complexity and limiting generalizability. Variability between institutions likely reflects differences in patient populations, AVS protocols, and laboratory assays, all of which contribute to discrepancies and may prevent the adoption of universal thresholds. Despite optimization with institution-specific thresholds, other diagnostic metrics remained suboptimal, though specificity for right-sided disease was still high at 95.3% (95% CI 90–98%). Accordingly, the optimized Aldosterone_LAV_:cAldosterone_RAV_ ratio may be used as a decision-support tool when suggestive of right-sided dominance.

Several factors may explain differences between the original findings by Tuandam et al. and the results in our cohort. First, the sample size of analyzed patients was small (*n* = 68) [13]. Additionally, fewer than 25% of cases analyzed in the study had bilateral disease (*n* = 14), possibly skewing the cutoff values. Notably, only cases with successful bilateral AVS and imaging-concordant imaging were analyzed, while patients with discordant AVS and imaging findings were excluded. Prior studies have shown that up to half of AVS cases with confirmed primary aldosteronism demonstrate discordant imaging findings [7, 15, 16]. Multiple explanations include lesion size smaller than imaging resolution (e.g. microadenomas or focal hyperplasia) and inability for imaging to differentiate a functional aldosteronoma from an adrenal incidentaloma [5, 7]. Therefore, exclusion of imaging-discordant findings, which accounted for around one third of cases in the Tuandam et al. initial cohort, may have contributed to performance statistics that differ when applied to a broader, less selective population. This is supported by our subgroup analysis: restricting our cohort to patients with a dominant adrenal mass concordant with the AVS-determined subtype raised accuracy with the published thresholds from 43.3 to 79.4%, approaching the originally reported performance (Supplementary Table S1). The operative variable appears to be the resulting proportion of bilateral disease, which fell from 54% in the full cohort to 11% in this subgroup. By contrast, when the concordant group also included patients with bilateral disease and no adrenal mass on imaging, accuracy fell to 47.7% despite imaging and AVS remaining in agreement. This suggests that the high accuracy observed among imaging-concordant cases reflects the exclusion of bilateral disease rather than any benefit of concordance itself. Finally, the equations published by Tuandam et al. assume symmetric cortisol secretion from both adrenal glands, yet asymmetric cortisol co-secretion may occur in up to 18% of patients [24], limiting the model’s applicability in such cases and potentially invalidating the underlying assumption in diseased glands.

A prior study by Pasternak et al. proposed an alternate index, aldosterone:cortisol ratio of the left adrenal vein compared with the inferior vena cava (LAV/IVC) with promising significant results. Unfortunately, this also required recalibration and institution optimization by Tuandam et al. for only marginally better performance at a second center [13, 14, 22, 23]. Zibar Tomsic et al. also derived institution-specific cutoffs, using the adrenal aldosterone:cortisol and adrenal vein:IVC ratios. These were highly specific for unilateral disease but performed poorly in bilateral disease (AV/IVC < 0.68: 28.8% sensitivity, AUC 0.646), despite being based on measured rather than calculated aldosterone values [25]. These findings demonstrate that, to use a proposed calculated index, institution-optimized thresholds must be derived. While an effort to find a calculated index is reasonable given the difficulty of right-sided adrenal vein cannulation, most calculated indices have failed to perform satisfactorily at outside institutions, despite optimization [13, 14, 26–28]. This implies that no calculated index has been robust enough to compare to technically successful AVS results. The difficulty therefore appears to lie in interpreting incomplete AVS itself, not in the particular formula used. Despite the valiant efforts of multiple groups, including Tuandam et al., to identify a reliable surrogate index, successful AVS cannulation remains the gold standard and interventional radiologists should prioritize technical optimization.

There are several techniques and workflow considerations to help facilitate right adrenal catheterization and sampling, including but not limited to a thorough understanding of right adrenal venographic patterns[21], having a variety of catheter shapes in inventory to account for different angles of right adrenal vein insertion on the IVC, performing preprocedure CT venogram to assess for anatomic variants, avoiding oversedation to minimize deep respirations, and using cone beam CT [29] or intraoperative rapid cortisol assays [30] to confirm catheterization prior to sampling.

This study has several limitations such as it was retrospective and conducted at a single-center tertiary-care center. Although the Aldosterone_LAV:cAldosterone_RAV ratio was calculated using only left adrenal vein and inferior vena cava values, as it would be when right adrenal vein sampling fails, all patients in this study had technically successful bilateral sampling. Patients in whom right cannulation fails may differ in ways that affect performance, but validation in that population is precluded by the absence of a reference standard. Five interventional radiologists performed the procedures, introducing operator variability. Additionally, not all patients underwent confirmatory testing prior to AVS procedure, though in these cases there remained high clinical suspicion for primary aldosteronism. Standard AVS interpretation, rather than postoperative outcome, served as the reference standard. Although a subset of patients underwent adrenalectomy, postoperative biochemical and clinical follow-up was incomplete. Outcome data are also only for patients with unilateral disease who elected for surgery. Restricting the analysis to that subgroup would have introduced selection bias this study sought to avoid and would have provided no reference standard for the patients with bilateral disease. Confirmation against postoperative biochemical remission would nonetheless strengthen the reference standard and warrants evaluation in future work.

Furthermore, only ACTH-stimulated AVS cases were included, so these findings may not generalize to institutions using non-stimulated or alternative stimulation protocols. This study also assesses the predictive value of the published equations under the assumption of symmetric cortisol secretion bilaterally and therefore may not be valid in patients with mild autonomous cortisol secretion, which could not be excluded in all patients as dexamethasone suppression testing was not performed universally.

## Conclusion

In our external validation cohort, the Aldosterone_LAV_:cAldosterone_RAV_ ratio had suboptimal performance for disease subtyping, even with institution-optimized thresholds. This index may be used as decision-support adjunct only in limited contexts such as implied right-sided dominant disease given its high specificity in this scenario but should not replace standard AVS interpretation and must be institution-specific. Therefore, these results reinforce the critical importance of good technique when performing AVS to achieve technically successful bilateral cannulation and diagnostic samples.

## Supplementary Information

Supplementary Material 1: Supplementary Table S1. Diagnostic accuracy of the calculated index across imaging-concordance and disease-subtype subgroups.

## Data Availability

The datasets generated and/or analyzed during the current study are not publicly available due institutional restrictions related to retrospective electronic medical record chart review data but are available from the corresponding author on reasonable request.

## Abbreviations

AVS: Adrenal vein sampling
IVC: Inferior vena cava
LAV: Left adrenal vein
RAV: Right adrenal vein
