# Supplementary material for: When “right” goes wrong: poor accuracy of calculated right adrenal vein aldosterone levels in subtyping primary aldosteronism
Source: CVIR Endovasc. 2026 Sep 4;9:114. doi: 10.1186/s42155-026-00769-6 (PMC13545160; doi:10.1186/s42155-026-00769-6)
Supplement: Supplementary file 1 [file 42155_2026_769_MOESM1_ESM.docx]

**Supplementary Material**

|  |  |  | **Published Threshold** | |  | **Optimized Threshold** | |  |
| --- | --- | --- | --- | --- | --- | --- | --- | --- |
| **Subgroup** | **n** | **Bilateral disease, n (%)** | **Correct** | **Accuracy** | **95% CI** | **Correct** | **Accuracy** | **95% CI** |
|  |  |  |  |  |  |  |  |  |
| **Full cohort** | 164 | 88 (53.7%) | 71 | 43.3% | 35.9–50.9% | 108 | 65.9% | 58.3–72.7% |
|  |  |  |  |  |  |  |  |  |
| Imaging-concordant | 109 | 53 (48.6%) | 52 | 47.7% | 38.6–57.0% | 73 | 67.0% | 57.7–75.1% |
| Imaging-discordant | 55 | 35 (63.6%) | 19 | 34.5% | 23.4–47.7% | 35 | 63.6% | 50.4–75.1% |
|  |  |  |  |  |  |  |  |  |
| Concordant, visible mass only | 63 | 7 (11.1%) | 50 | 79.4% | 67.8–87.5% | 56 | 88.9% | 78.8–94.5% |
| Discordant, visible mass only | 42 | 35 (83.3%) | 10 | 23.8% | 13.5–38.5% | 25 | 59.5% | 44.5–73.0% |
|  |  |  |  |  |  |  |  |  |
| Unilateral cases only | 76 | 0 (0.0%) | 64 | 84.2% | 74.4–90.7% | 69 | 90.8% | 82.2–95.5% |
| Bilateral cases only | 88 | 88 (100.0%) | 7 | 8.0% | 3.9–15.5% | 39 | 44.3% | 34.4–54.7% |

**Supplementary Table 1. Diagnostic accuracy of the calculated index across imaging-concordance and disease-subtype subgroups.**

Table Note: Accuracy represents the proportion of correctly classified cases using the published and institution-optimized thresholds. Imaging concordance was defined as a dominant adrenal mass on the side of AVS lateralization for unilateral disease, and as either bilateral adrenal masses or no dominant adrenal mass for bilateral disease; all other combinations were classified as imaging-discordant. Visible-mass only rows are restricted to patients with a dominant adrenal mass on imaging.
